# Deletion of bone marrow myeloperoxidase attenuates chronic kidney disease accelerated atherosclerosis

**DOI:** 10.1074/jbc.RA120.014095

**Published:** 2020-12-03

**Authors:** Anna V. Mathew, Lixia Zeng, Kevin B. Atkins, Kiana N. Sadri, Jaeman Byun, Hideaki Fujiwara, Pavan Reddy, Subramaniam Pennathur

**Affiliations:** 1 Division of Nephrology, Department of Medicine, University of Michigan, Ann Arbor, Michigan, USA; 2 Division of Hematology-Oncology, Department of Medicine, University of Michigan, Ann Arbor, Michigan, USA; 3 Department of Molecular and Integrative Physiology, University of Michigan, Ann Arbor, Michigan, USA

**Keywords:** chronic kidney disease, atherosclerosis, myeloperoxidase, macrophages, oxidized amino acids, CVD, cardiovascular disease, CKD, chronic kidney disease, ESI, electron spray ionization, HFD, high-fat diet, iPTH, intact parathyroid hormone, LDL, low-density lipoprotein, MPO, myeloperoxidase, SAA, serum amyloid A

## Abstract

Increased myeloperoxidase (MPO) expression and activity are associated with atherosclerotic disease in patients with chronic kidney disease (CKD). However, the causal relationship between MPO and the development and progression of atherosclerosis in patients with CKD is unknown. Eight-week-old male low-density-lipoprotein-receptor–deficient mice were subjected to 5/6 nephrectomy, irradiated, and transplanted with bone marrow from MPO-deficient mice to induce bone marrow MPO deletion (CKD-bMPOKO) or bone marrow from WT mice as a control to maintain preserved bone marrow MPO(CKD-bMPOWT). The mice were maintained on a high-fat/high-cholesterol diet for 16 weeks. As anticipated, both groups of mice exhibited all features of moderate CKD, including elevated plasma creatinine, lower hematocrit, and increased intact parathyroid hormone but did not demonstrate any differences between the groups. Irradiation and bone marrow transplantation did not further affect body weight, blood pressure, creatinine, or hematocrit in either group. The absence of MPO expression in the bone marrow and atherosclerotic lesions of the aorta in the CKD-bMPOKO mice was confirmed by immunoblot and immunohistochemistry, respectively. Decreased MPO activity was substantiated by the absence of 3-chlorotyrosine, a specific by-product of MPO, in aortic atherosclerotic lesions as determined by both immunohistochemistry and highly sensitive LC-MS. Quantification of the aortic lesional area stained with oil red O revealed that CKD-bMPOKO mice had significantly decreased aortic plaque area as compared with CKD-bMPOWT mice. This study demonstrates the reduction of atherosclerosis in CKD mice with the deletion of MPO in bone marrow cells, strongly implicating bone-marrow-derived MPO in the pathogenesis of CKD atherosclerosis.

Chronic kidney disease (CKD) is prevalent in 15% of the US population, and currently, nearly 650,000 of these individuals with CKD need renal replacement therapies (1). Cardiovascular disease (CVD) continues to be the leading cause of mortality in patients with CKD (1, 2, 3, 4, 5, 6, 7). The risk of CVD in patients with CKD increases as their renal function worsens, with an average risk of 10 to 40 times that of the healthy population (8, 9, 10, 11, 12). CKD-accelerated atherosclerosis is the most prevalent CVD linked to CKD, occurring in ∼40% of CKD patients older than 65 years of age. Although patients with CKD often have traditional risk factors for atherosclerosis, many CKD-specific risk factors are also implicated in CKD-accelerated atherosclerosis, including oxidative stress, inflammation, endothelial dysfunction, uremic toxins, anemia, heart failure, bone mineral disease, and an altered renin–angiotensin system (5, 13, 14, 15, 16, 17). Levels of oxidized low-density lipoprotein (LDL) are tenfold higher in patients with CKD than in healthy control patients (18, 19). The oxidized LDLs present in atherosclerotic lesions in CKD patients have an increased affinity for macrophage scavenger receptors and rapidly accumulate in the macrophages creating foam cells (14, 20, 21, 22, 23, 24, 25, 26, 27). CKD-accelerated atherosclerosis is characterized by more extensive and advanced lesions with fibrotic and calcified components than atherosclerosis that is not associated with CKD (28, 29).

Myeloperoxidase (MPO), a heme peroxidase enzyme, is expressed in neutrophils and monocytes (30). Inflammatory states trigger MPO release and can cause oxidative tissue injury. Evidence of MPO activity is present in all stages of human atherosclerotic lesions (31, 32, 33). While macrophages in early fatty streaks have little to no MPO expression, MPO expressing macrophages are present in eroded and ruptured plaques (34). MPO generates hypochlorous acid from hydrogen peroxide, which then chlorinates tyrosine residues to create 3-chlorotyrosine (33). Similarly, MPO can generate 3-nitrotyrosine from nitrogen dioxide radicals (NO_2_^•^) (35, 36). MPO is also implicated in vascular dysfunction by reducing the availability of nitric oxide and promoting the formation of reactive nitrogen species (37, 38).

MPO expression and evidence of MPO activity are present in the proximity of macrophages in atherosclerotic lesions from patients (32, 33, 39, 40, 41); however, a causal role of macrophage MPO in atherosclerosis has not been established. More importantly, elevated plasma MPO levels predict the risk of cardiovascular events and mortality in patients with unstable angina, chest pain, acute coronary syndrome, or peripheral arterial disease (42, 43, 44). Systemic levels of 3-nitrotyrosine have also been found to be higher among patients with CVD as compared with individuals with healthy arteries (45). Clinical studies on patients with CKD revealed that higher MPO levels and higher MPO activity were associated with worsening CKD stage and worsening CVD (46, 47, 48, 49, 50). An MPO-derived protein modification, carbamylation, is also elevated in CKD patients with CVD (51, 52, 53, 54). Additionally, plasma MPO levels and MPO oxidation products are elevated in patients in autoimmune diseases, such as rheumatoid arthritis and systemic lupus erythematosus, both of which carry an increased risk of CVD (55, 56). Therefore, strong associative evidence links MPO, CKD, and CVD; however, these clinical studies do not prove causation.

Genetic manipulation of MPO in rodent models has yielded conflicting results because mouse models of atherosclerosis in the absence of CKD have very low baseline MPO oxidation products detected in atheroma (57). MPO-deficient mice exhibit exaggerated atherosclerosis, but mice with MPO overexpression in the macrophages from a human transgene also exhibit increased atherosclerosis (57, 58). Notably, MPO is highly expressed in unstable plaques and both genetic and pharmacological inhibition of MPO increased plaque stability (59). In a recent study, we demonstrated robust MPO expression and catalytic activity in a mouse model of CKD that exhibits many biochemical features of CKD following 5/6 nephrectomy in LDL receptor knockout (LDLr^−/−^) mice (28). Elevated MPO oxidation products were colocalized with macrophages in the atherosclerotic lesions in this model, raising the possibility that macrophage MPO may play a role in the progression of atheroma.

To test a causal role of bone-marrow-derived MPO in CKD-accelerated atherosclerosis, we created a mouse model of CKD on the LDLr^−/−^ background with bone marrow cell–specific modulation of MPO expression. Using irradiation followed by bone marrow transplant from WT or MPO-knockout mice, we created a bone marrow cell–specific presence or absence of MPO expression in atherosclerosis prone LDLr^−/−^ mouse model of CKD. We demonstrate that, despite features of CKD in the mice, the absence of MPO in the bone marrow cells caused a significant decrease in atherosclerotic burden without a change in vascular reactivity. These studies support a causal role for bone-marrow-derived MPO in the pathogenesis of CKD-accelerated atherosclerosis.

## Results

### Nephrectomized LDLr^−/−^ mice with bone marrow cell–specific MPO modulation have features of moderate CKD

C57BL/6 LDLr^−/−^ mice were subjected to 5/6 nephrectomy and, at 8 weeks of age, underwent whole-body irradiation and bone marrow transplant with bone marrow from WT mice (CKD-bMPOWT) or MPO knockout mice (CKD-bMPOKO). Within 2 to 4 weeks, the mice recovered their body weight and regained hematocrit levels (Table 1). The absence of MPO in the bone marrow of the CKD-bMPOKO mice was confirmed with immunoblot of MPO expression (Fig. S1). The mice were then maintained on a high-fat/high-cholesterol diet (HFD) for 16 weeks.

**Table 1:** Biological characteristics of the mouse models

Variables	N (each group)	CKD-bMPOWT	CKD-bMPOKO	*p*-value
Bodyweight (gm)				
Baseline	10	22.5 ± 1.4	22.8 ± 1.8	0.67
Post-BMT	10	24.2 ± 1.4	24.2 ± 1.7	0.99
16 weeks (HFD)	10	29.4 ± 2.9^a^	28.1 ± 2.4^a^	0.41
Systolic BP (mmHg)	5	161.96 ± 14.6	158.9 ± 13.8	0.84
Diastolic BP(mmHg)	5	139.56 ± 14.5	132.4 ± 12.6	0.46
Creatinine (mg/dl)				
Pre-BMT	5	0.25 ± 0.04	0.31 ± 0.12	0.36
Post-BMT	5	0.32 ± 0.07	0.33 ± 0.05	0.79
16 weeks (HFD)	5	0.22 ± 0.02^a^	0.24 ± 0.05^a^	0.56
Hematocrit (%)				
Baseline	10	50.9 ± 1.1	51.2 ± 1.8	0.65
Post-BMT	10	51.6 ± 2.5	52.4 ± 4.2	0.63
16 weeks (HFD)	10	51.3 ± 4.5	51.5 ± 3.0	0.94
Serum calcium (mg/dl)	8	12.8 ± 2.5	12.5 ± 2.6	0.8
Serum phosphorus (mg/dl)	8	20.48 ± 4.33	24.6 ± 4.9	0.1
Intact parathyroid hormone (pg/ml)	5	17.9 ± 14.0	32.3 ± 37.1	0.44

BMT, bone marrow transplant; BUN, blood urea nitrogen; CKD-bMPOKO, irradiated 5/6 nephrectomized LDLr^−/−^ mice with bone marrow from MPO knockout mice; CKD-bMPOWT, irradiated control 5/6 nephrectomized LDLr^−/−^ mice with bone marrow from WT donor mice; HFD, high-fat/high-cholesterol diet; iPTH; intact parathyroid hormone.

a *p*-value < 0.05 as compared with mice in the same group at previous time points.

Mice from both groups gained significant body weight over time (*p* < 0.05; Table 1). There was no difference in body weight between the two groups of mice either at baseline after 5/6 nephrectomy, or post–bone marrow transplant, or at the end of 16 weeks on an HFD. There were also no differences in systolic or diastolic blood pressure between the two groups.

Serum creatinine levels in the CKD-bMPOWT and CKD-bMPOKO mice were elevated, confirming renal insufficiency in the CKD model. Serum creatinine was not different between the two groups, either before or after the bone marrow transplant (*p* > 0.05; Table 1), suggesting no independent effect of bone marrow transplant on renal function. Serum creatinine did not differ after 16 weeks on an HFD, and the mice in both groups displayed other features of renal insufficiency, such as anemia and hyperparathyroidism. Hematocrit did not differ between the two groups after 5/6 nephrectomy, after bone marrow transplant, or after 16 weeks of an HFD. There were also no differences in serum calcium, phosphorus, or intact parathyroid hormone levels between the two groups (Table 1).

### Effects of CKD, HFD, and bone marrow cell–specific MPO deletion on plasma lipid levels

**Figure 1:**
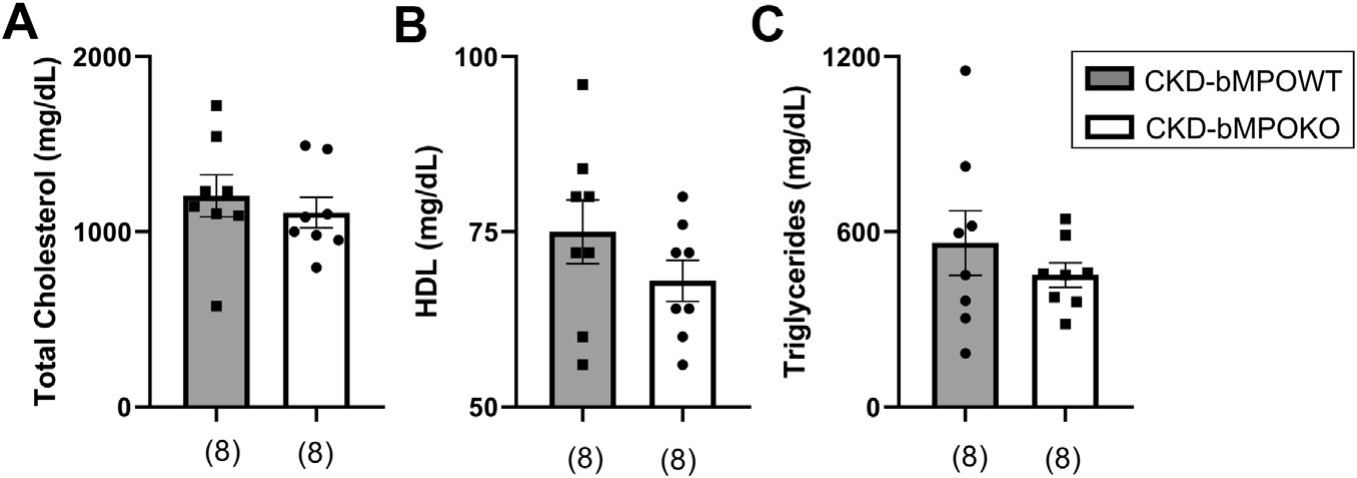
**Bone marrow MPO deficiency in 5/6 nephrectomized mice does not alter lipid profiles.** Deletion of bone marrow MPO (bMPOKO) does not alter. *A*, total cholesterol, *B*, high-density lipoprotein (HDL), or *C*, triglycerides in 5/6 nephrectomized (CKD) mice as compared with CKD mice with preserved bone marrow MPO (CKD-bMPOWT) after 16 weeks on a high-fat/high-cholesterol diet. All *p* values >0.05.

Both groups of mice had dyslipidemia after consuming an HFD for 16 weeks. However, there were no differences in total cholesterol, high-density lipoprotein cholesterol, or triglycerides in the mice with bone marrow cell–specific deletion of MPO compared with the mice expressing MPO in the bone marrow (Fig. 1, *A*–*C*). Therefore, changes in bone marrow MPO expression do not alter lipid levels in this model of CKD.

### Effects of bone marrow cell–specific MPO deletion on vascular reactivity in CKD mice

**Figure 2:**
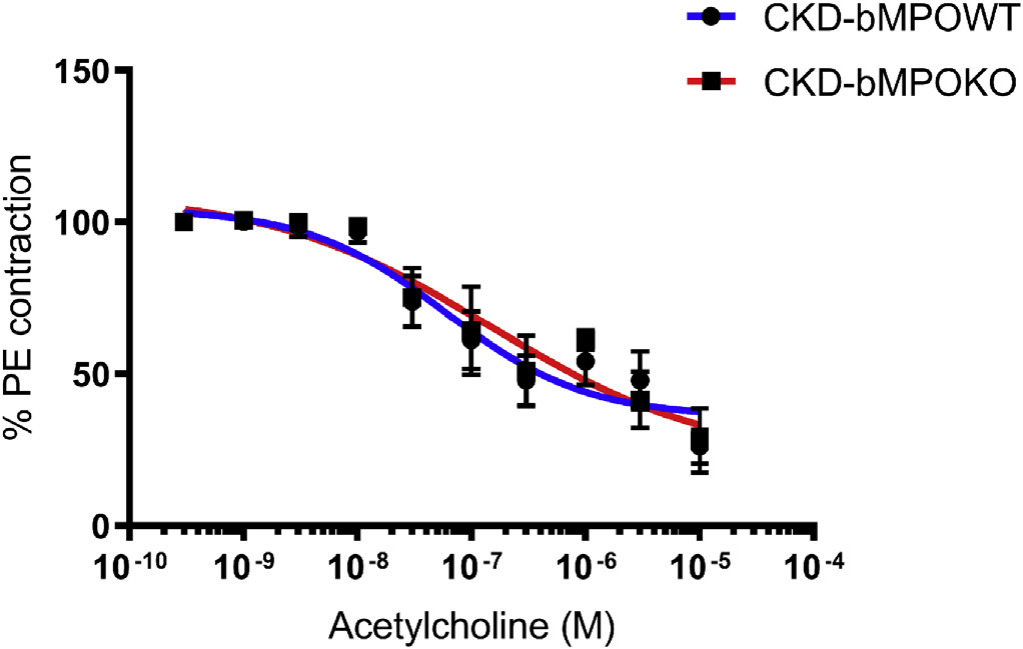
**Bone marrow deletion of MPO in mice with 5/6 nephrectomy on a high-fat diet does not affect vascular reactivity.** Response relaxation curve of response to acetylcholine of aortic rings precontracted with phenylephrine from 5/6 nephrectomized mice with bone marrow MPO deficiency (CKD-bMPOKO) or WT bone marrow expression of MPO (CKD-bMPOWT) on high-fat diet/high-cholesterol diet for 16 weeks n = 5/group. All *p* values >0.05.

Given the associative evidence linking MPO, atherosclerosis, and vascular responses, we assessed the cholinergic responsiveness of mouse aortic rings in mice with and without bone-marrow-derived MPO. The aortic rings from CKD-bMPOKO and CKD-bMPOWT mice both relaxed with increasing concentrations of acetylcholine. Maximum responses (Emax) for the aortic rings of CKD-bMPOWT and CKD-bMPOKO mice were not significantly different (*p* = 0.68; Fig. 2). These data suggest that bone marrow MPO deletion does not alter vascular dysfunction in this model.

### Assessment of MPO and tyrosine oxidation products in atherosclerotic lesions

**Figure 3:**
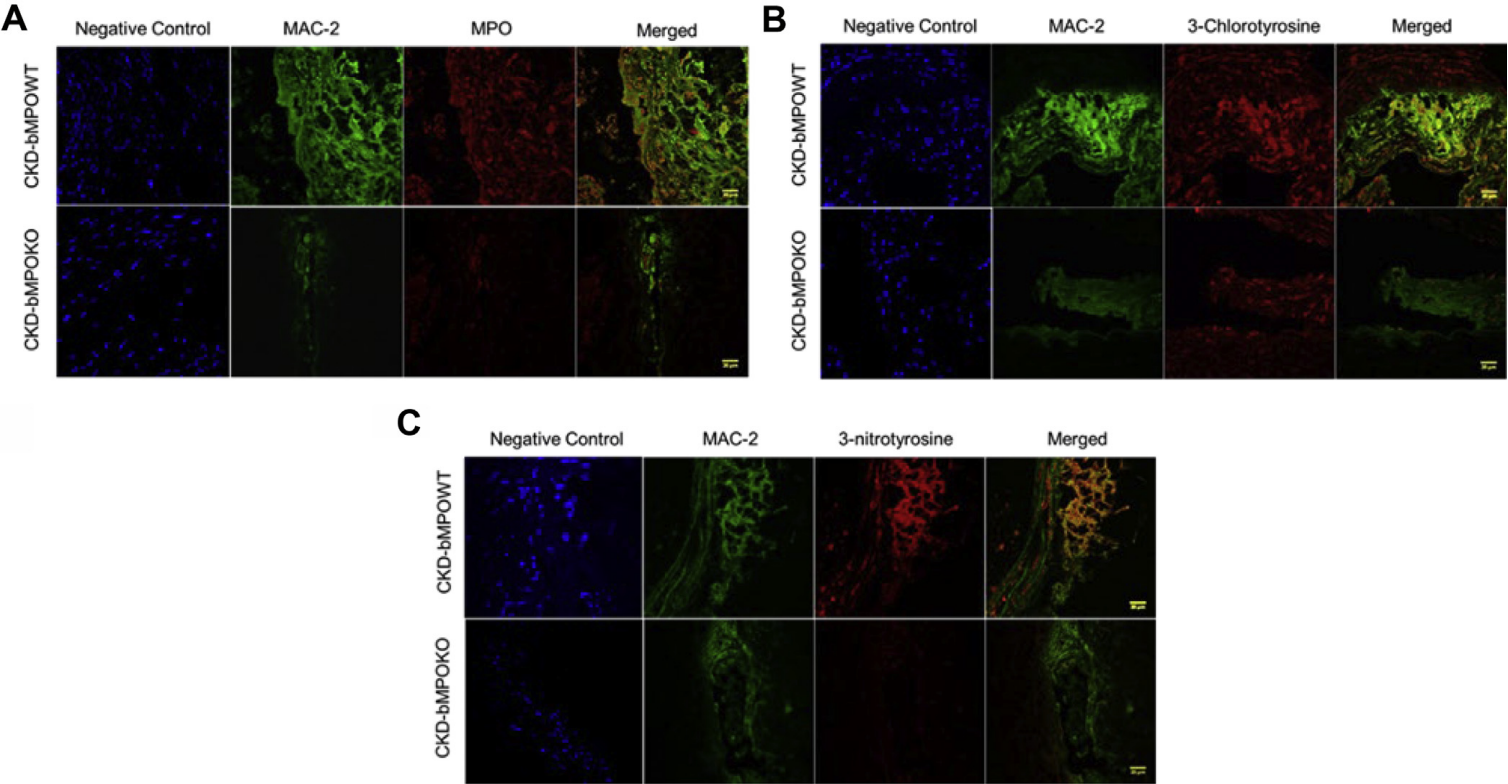
**MPO and its oxidation products colocalize with macrophages and are decreased in the vascular wall of 5/6 nephrectomized mice with bone marrow deletion of MPO.** Representative immunofluorescence and double labeling in the aortic cross sections from male LDLr^−/−^ 5/6 nephrectomized mice after 16 weeks on a high-fat/high-cholesterol diet with bone marrow deletion of MPO (CKD-bMPOKO) or intact bone marrow MPO expression (CKD-bMPOWT). Staining for Mac-2 (macrophage marker, green) and red staining for (*A*) MPO, (*B*) 3-chlorotyrosine, and (*C*) 3-nitrotyrosine, are shown. Yellow staining (MERGE) indicates the colocalization of MPO and oxidation markers with Mac-2. Also represented are the negative controls with DAPI (blue) staining only. Scale bars represent 20 μm.

MPO oxidizes tyrosine moieties in proteins to generate oxidized tyrosines. We examined MPO expression and the presence of oxidized tyrosines using immunohistochemistry in our CKD mouse model with bone marrow from WT or MPO-knockout mice. After 16 weeks on an HFD, MPO expression was observed in atherosclerotic lesions in aortic cross sections from CKD-bMPOWT mice but not in aortic lesions in CKD-bMPOKO mice (Fig. S2). Similarly, immunohistochemical staining for MPO-derived tyrosine modifications using specific antibodies for 3-chlorotyrosine and 3-nitrotyrosine demonstrated robust staining in the CKD-bMPOWT mice but not in the CKD-bMPOKO mice (Fig. S3). Furthermore, double staining for macrophage marker—Mac-2 and MPO (Fig. 3*A*), and MPO oxidation products—3-chlorotyrosine (Fig. 3*B*), and 3-nitrotyrosine (Fig. 3*C*) revealed decreased colocalization of MPO and oxidation markers in the macrophages in lesions from CKD-bMPOKO mice as compared with CKD-bMPOWT mice.

**Figure 4:**
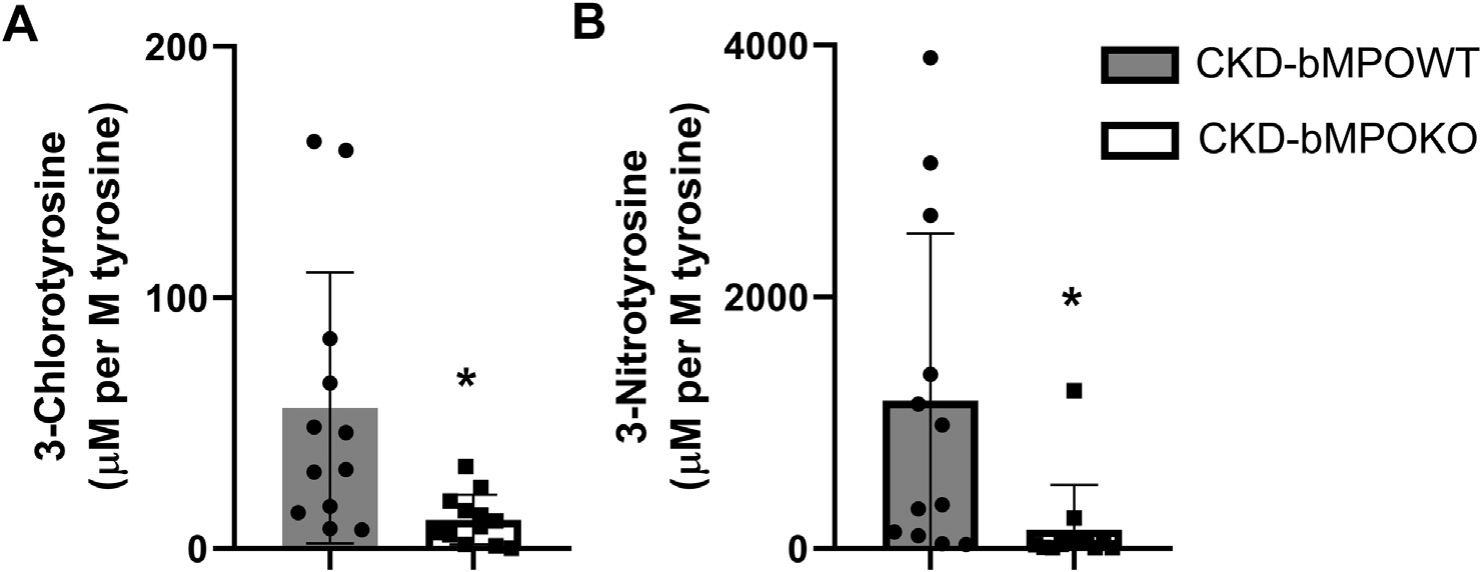
**Bone marrow MPO deficiency in 5/6 nephrectomized mice on a high-fat/high-cholesterol diet decreases MPO-dependent oxidation in atherosclerotic lesions.** Mass spectrometric quantification of oxidized amino acids in aortic proteins from irradiated 5/6 nephrectomized LDLr ^−/−^ mice (CKD) mice with WT bone marrow (CKD-bMPOWT) and with bone marrow MPO deficiency (CKD-bMPOKO) after 16 weeks on a high-fat/high-cholesterol diet. *A*, 3-chlorotyrosine and *B*, 3-nitrotyrosine expressed as ratios to the precursor amino acid, tyrosine, in μmol/mol (n= 12 each). ∗*p* < 0.05.

To confirm the activity of MPO in the atherosclerotic lesions, we utilized stable-isotope-dilution MS, a highly sensitive and specific method to quantify the levels of the 3-chlorotyrosine and 3-nitrotyrosine protein tyrosine oxidative modifications in aortic lesions. In contrast to qualitative immunoassays, MS provides accurate quantitative data that is essential to demonstrate enzyme activity. After 16 weeks on an HFD, average 3-chlorotyrosine levels were markedly increased in the aortic tissue of the CKD-bMPOWT mice as compared with the CKD-bMPOKO mice (56.1 ± 54 *versus* 11.5 ± 10 μM per M of tyrosine; *p* = 0.004; Fig. 4*A*). Hence the absence of bone marrow MPO expression led to a marked reduction in 3- chlorotyrosine, a specific MPO product, in atherosclerotic lesions of CKD mice. Similarly, 3-nitrotyrosine levels were also decreased in the CKD-bMPOKO mice (1174 ± 1332 μM per M of tyrosine) as compared with CKD-bMPOWT mice (150.4 ± 354 μM per M of tyrosine, *p* = 0.002; Fig. 4*B*). These data strongly suggest that tyrosine oxidation products are decreased in atherosclerotic aortic tissue when the macrophages in the atherosclerotic tissue lack MPO.

### Bone marrow cell–specific MPO deletion decreases atherosclerosis in CKD mice

**Figure 5:**
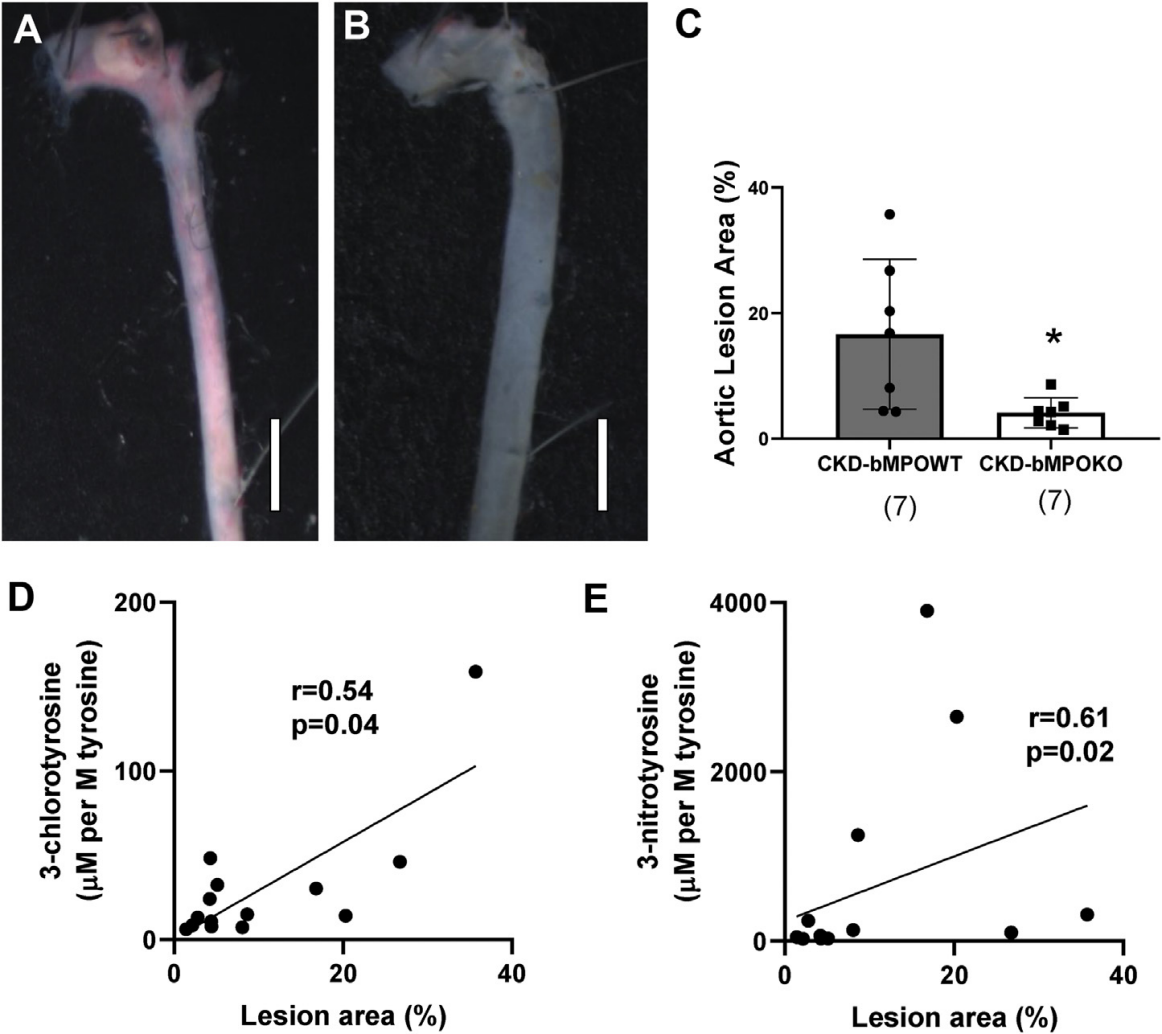
**Atherosclerosis is reduced in 5/6 nephrectomized mice with bone marrow MPO deficiency in proportion to the MPO-dependent oxidation in atherosclerotic lesions.** Oil red staining and morphometry of *en face* aorta from irradiated 5/6 nephrectomized LDLr ^−/−^ mice (CKD) after 16 weeks on a high-fat/high-cholesterol diet. *A*, CKD mice with WT bone marrow (CKD-bMPOWT). *B*, CKD mice with bone marrow MPO deficiency (CKD-bMPOKO). Scale bar represents 2 mm. *C*, analysis of total atherosclerotic lesion area as a ratio (ratio of Oil red O stained area to the total surface area of the *en-face* section of the aortic tree expressed as a percentage) reveals decreases in the area occupied by atherosclerotic lesions in the CKD-bMPOKO mice as compared with the CKD-bMPOWT mice. Panels *D* and *E* show correlation of oxidized amino acids in aortic proteins and stained atherosclerotic area (n = 14).∗*p* < 0.05.

*En face* analysis of the area occupied by atherosclerotic lesions in the aortic tree was performed to quantify atherosclerosis. Oil red O staining of the aortic tree and subsequent comparative morphometry revealed decreased atherosclerotic lesions in irradiated CKD mice with bone marrow deletion of MPO expression (CKD-bMPOKO) compared with irradiated CKD mice with donor marrow from WT mice (CKD-bMPOWT; Fig. 5, Fig. S4). Panels 5, A–B show representative *en face* Oil red O staining from CKD-bMPOWT and CKD-bMPOKO groups, respectively, while Panel 5C demonstrates the comparative morphometry of the stained aortic areas between the two groups. Levels of 3-chlorotyrosine and 3-nitrotyrosine correlated with lesion area (%) using Spearman correlation (r = 0.51 and r = 0.61 respectively; *p* < 0.05; Panels 5, D–E). The significant correlation between MPO products (3-chlorotyrosine and 3-nitrotyrosine) and lesion area confirms a direct relationship between MPO activity and degree of CKD atherosclerosis.

**Figure 6:**
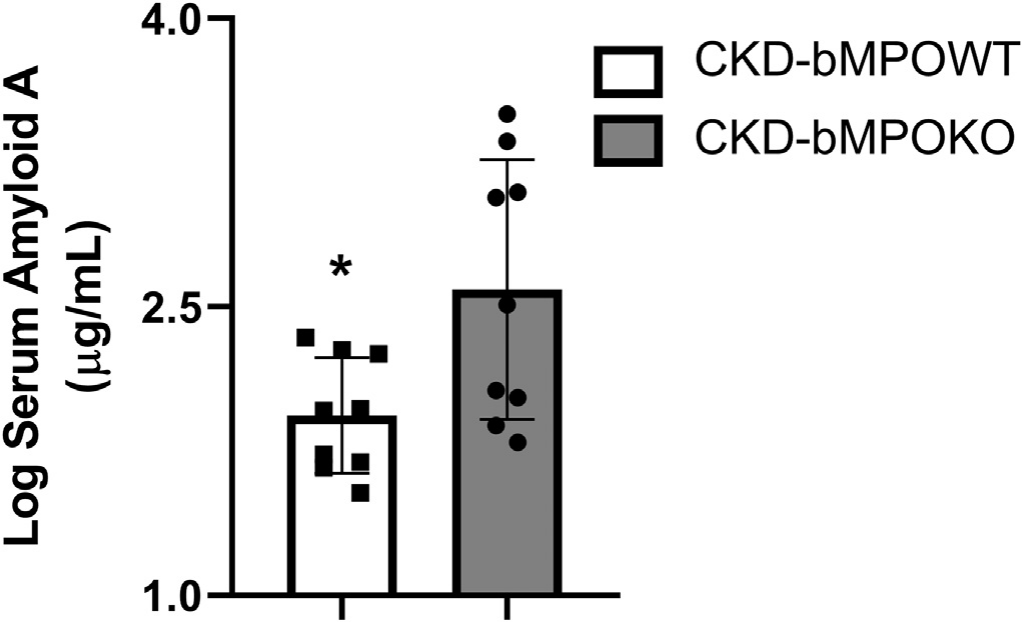
**Serum Amyloid A levels are increased in 5/6 nephrectomized mice with bone marrow MPO deficiency.** Serum amyloid A (SAA) protein levels are increased with bone marrow MPO deficiency in irradiated 5/6 nephrectomy mice (CKD) LDLr ^−/−^ mice after 16 weeks on a high-fat/high-cholesterol diet (n= 9 each group; ∗*p* < 0.05).

We also measured the proinflammatory acute phase reactant Serum Amyloid A protein (SAA) in both groups to test whether altered immune responses secondary to bone marrow MPO deficiency could account for differences in the phenotype. Surprisingly, the SAA levels were elevated in the CKD-bMPOKO mice despite decreased atherosclerosis, suggesting that MPO expression was the primary driver of this phenotype (*p* < 0.05. Fig. 6).

## Discussion

MPO-mediated oxidation is associated with increased atherosclerotic disease and vascular dysfunction with decreased renal function—in both animal and human models (28, 60, 61). The current study is the first to demonstrate a causal relationship between bone-marrow-derived MPO and CKD-accelerated atherosclerosis. We deleted bone marrow MPO on the atherosclerosis-prone CKD mouse model. The mice did not demonstrate any changes to the blood pressure, renal function, or lipid profile in response to MPO deficiency in bone marrow cells. Despite this, bone marrow MPO deficiency markedly decreased the atherosclerotic burden in CKD mice. As anticipated, MPO deficiency decreased the production of oxidized tyrosine products in the arterial walls of the mice with CKD, demonstrating decreased MPO activity in the artery wall.

LDLr^−/−^ mice with 5/6 nephrectomy demonstrate a consistent decrease in renal function and reflect atherosclerosis accelerated by moderate CKD in humans (28, 62). Our previous study demonstrated extensive atherosclerosis with fibrosis and luminal narrowing in the CKD mice compared with controls after 12 and 24 weeks on an HFD and increased macrophage-derived MPO activity (28). Here, we used irradiation and subsequently transplanted the CKD mice with bone marrow cells derived from MPO deficient and WT mice to modulate MPO expression in the bone marrow cells. Consistent with our prior work, these mice had many features of CKD, including elevated creatinine, anemia, increased intact parathyroid hormone, with no evidence of hypercalcemia (28, 62). Significantly, irradiation and bone marrow transplantation did not alter any biochemical or physiologic characteristics of the model. The CKD mice with bone marrow MPO deletion had decreased atherosclerosis despite a lack of differences in traditional risk factors for atherosclerosis. To our knowledge, this is the first mouse model with bone marrow MPO modulation, which demonstrates a role for MPO in CKD-accelerated atherosclerosis. MPO expression and oxidation products colocalized with macrophages suggesting a crucial role for macrophage MPO in the artery wall in the pathogenesis of CKD atherosclerosis (23, 28). However, it is also possible the reduction of MPO in other cell types such as neutrophils and monocytes could also have contributed to the decrease in atherosclerosis observed in the MPO-deficient state.

In contrast to our work on CKD atherosclerosis, the role of MPO in mouse models of atherosclerosis with preserved renal function has been controversial (28). LDLr^−/−^ mice with preserved renal function did not demonstrate increased 3-chlorotyrosine levels in atherosclerotic lesions, suggesting that MPO may not be catalytically active in lesions as a result of a low-level of MPO expression in phagocytic cells in contrast to increased MPO expression and activity demonstrated in human atherosclerotic lesions (57). MPO deficiency paradoxically increased atherosclerosis in the LDLr^−/−^ mice with preserved renal function suggesting unknown off-target effects for MPO (57). On the other hand, overexpression of human MPO transgene in macrophages increased atherosclerosis in mice with normal renal function, suggesting that excess macrophage MPO could worsen atherosclerosis (58). Besides, the use of MPO inhibitors decreased necrosis and inflammation of the atherosclerotic lesions and increased plaque stability and neointima formation (59, 63, 64). Therefore, both MPO and alternate non-MPO pathways may play a role in non-CKD atherosclerosis.

MPO is present in atherosclerotic lesions, and MPO-derived oxidants propagate oxidative damage both in macrophages and in extracellular sites. Our studies indicate that MPO and oxidative markers colocalize with lesional macrophages and their surrounding extracellular space (65, 66). The vast majority of cell-associated MPO immune reactivity is present in macrophages, and most of the extracellular MPO remains juxtaposed with macrophages (Fig. 4). While 3-chlorotyrosine colocalized with macrophages, there was also staining of extracellular modified proteins. These observations are consistent with the presence of extracellular MPO and/or the ability of MPO-generated HOCl to generate long-lived reactive intermediates such as chloramines, which can diffuse extracellularly to chlorinate proteins and generate 3-chlorotyrosine. Similarly, immunostaining for 3-nitrotyrosine and MPO demonstrates virtually identical patterns suggesting that extracellular matrix proteins can also be nitrated. Such damage to extracellular matrix proteins, such as fibronectin, can further propagate inflammation (67, 68).

Though 3-chlorotyrosine is very specific for MPO activity, it is produced in low levels *in vivo*, in part related to low reaction rate of hypochlorous acid with tyrosine residues as well as the possible further metabolism of 3-chlorotyroisne in biological systems (69, 70). Therefore, 3-chlorotyrosine may not be fully quantitative of MPO enzymatic activity, and measurement of 2-chlorethidium from hydro ethidium injection to mice has been proposed as an alternate method to provide a better index of MPO activity (71, 72). However, 3-nitrotyrosine is derived from multiple pathways, some MPO related and others non-MPO-related, and hence it is not a specific marker of MPO activity (73). In our studies, the levels of 3-nitrotyrosine and 3-chlorotyrsoine correlate and are modulated by MPO levels, suggesting that MPO is the likely source of these oxidants (28).

MPO produced in the vessel wall consumes the vasodilator nitric oxide causing vascular dysfunction and produces nitrogen dioxide, a reactive nitrogen species. Nitric oxide can also combine with NADPH oxidase-generated superoxide (O_2_^•^) to produce the reactive nitrogen species peroxynitrite (ONOO−), causing nitration of proteins. Our earlier work suggested that asymmetric dimethylarginine, a potent inhibitor of endothelial nitric oxide synthase, is elevated in mice with CKD and is associated with atherosclerotic lesions (62). Therefore, multiple pathways contribute to the vascular dysfunction observed in the CKD atherosclerosis animal model, including atherosclerosis, creation of reactive nitrogen species, elevated asymmetric dimethylarginine, and consumption of the vasodilator nitric oxide (74). Rat models of CKD demonstrated decreased cholinergic responses in the presence of hypertension and elevated renin–angiotensinogen (75, 76). We demonstrated a dysfunctional vascular response in CKD atherosclerosis mice with increased vascular wall MPO expression and no evidence of hypertension (28). MPO inhibitors preserve endothelial function in mouse models of atherosclerosis with intact renal function by oxidizing soluble guanylyl cyclase resulting in improvement in plaque area, cytokine levels, and oxidative stress (59, 64, 77, 78). In contrast, in a study by Golubinskaya and colleagues, MPO knockout mice with intact renal function and without atherosclerosis or blood pressure changes had similar vascular responses to acetylcholine as the controls (79). Bone marrow deletion of MPO in CKD atherosclerotic mice in our study showed no evidence of improvement in vascular dysfunction despite the reduction in atherosclerotic burden as compared with CKD mice with intact bone marrow MPO. Therefore, the vascular dysfunction observed in CKD mice might be independent of atherosclerosis extent and bone marrow MPO and likely involves other CKD-specific factors that require further exploration in future studies. Alternatively, non-MPO-derived inflammation might be a more critical driver of endothelial dysfunction in CKD atherosclerosis. MPO is a critical protein regulating innate immunity and its deficiency as created in our model could alter inflammatory response between the two groups. One such proinflammatory acute phase reactant is SAA, which is linked to CVD and CVD mortality (80, 81). However, contrary to expectation, bone marrow MPO deficiency increased SAA levels in CKD mice. Despite the increased SAA levels, the mice had decreased atherosclerosis highlighting the critical role of MPO in CKD atherosclerosis.

Our study has some limitations. The creation of bone marrow MPO deficiency required exposing the CKD mice to whole-body irradiation, which could have independent effects on atherosclerosis and vascular dysfunction. Given that the CKD group with intact bone marrow MPO expression also underwent bone marrow transplantation, this issue is somewhat mitigated. Significantly, the mice recovered within 4 weeks after the bone marrow transplantation, as indicated by the unchanged body weight, hematocrit, and renal function. The donor mice used to create CKD-bMPOWT and CKD-bMPOKO were derived from a similar C57BL/6J background but were not littermates (derived from breeding MPO^+/−^ mice), which might contribute to minor genetic variation. We cannot, therefore, exclude this as a contributing factor for the observed phenotypic differences. As noted in our earlier work (28), antibodies directed against 3-chlorotyrosine and 3-nitrotyrosine have no cross-reactivity; however, in contrast to the 3-nitrotyrosine antibody, the antibody directed against 3-chlorotyrosine is not specific. Therefore, the 3-chlorotyrosine-based immunochemical studies have to be considered as qualitative. To mitigate this issue, we have used the more sensitive and specific mass-spectrometry-based measurements to confirm the immunohistochemistry studies.

In conclusion, bone marrow MPO deficiency decreased atherosclerosis in a CKD mouse model without changes in body weight, renal function, hypercholesterolemia, or blood pressure. This study supports our earlier work that demonstrated increased MPO expression in lesional macrophages in the arterial wall during CKD-accelerated atherosclerosis and is free from confounding differences in risk factors for atherosclerosis such as hyperglycemia, hypercholesterolemia, and hypertension. Overall, our study provides evidence of a causal role of bone-marrow-derived MPO in the pathogenesis of atherosclerosis in CKD mice and identifies MPO as a potential therapeutic target in CKD-accelerated atherosclerosis.

## Experimental procedures

### Mouse model of CKD accelerated atherosclerosis

All animal procedures were approved by the University of Michigan Committee on Use and Care of Animals. Five-week-old male C57BL/6 LDLr^−/−^mice (Jackson Lab, Bar Harbor, ME) were maintained with water *ad libitum* and a standard rodent diet (Lab Diet Hudson, NH) containing 28.5% protein, 13.5% fat, 58.0% carbohydrates by calories, and 200 ppm cholesterol. These mice were housed in a climate-controlled, light-regulated facility with a 12:12 h light-dark cycle. At age 5 weeks, the mice were subjected to 5/6 nephrectomy to model (CKD) by removing the whole right kidney first followed by surgical removal of 2/3 of the left kidney a week later. At 8 weeks of age, the mice underwent bone marrow transplantation. The mice were exposed to a single dose of 10 Gy radiation and within 24 h were administered 10 million bone marrow cells *via* the tail vein obtained from WT (C57BL/6J) or MPO knockout mice (B6.129X1-Mpotm1Lus/J, Jackson Lab, Bar Harbor, ME). This created preserved wild-type bone-marrow-derived MPO expression (CKD-bMPOWT) or bone marrow MPO deletion (CKD-bMPOKO), respectively. The mice were allowed 2 weeks of recovery during which they are maintained on the standard diet and acidic water. At 12 weeks of age (4 weeks after bone marrow transplant), the mice were fed a high-fat/high-cholesterol diet (HFD) containing 19.5% protein, 40.5% fat with 0.5% cholesterol by weight, and 40.0% carbohydrates (Harlan Teklad Laboratory, Winfield, Iowa). The mice in each group were maintained on the HFD for 16 weeks and evaluated in a blinded manner.

Hematocrit was measured using StatSpin CritSpin Micro-Hematocrit centrifuge with a digital hematocrit reader (Beckman Coulter, Inc, Brea, CA, USA). Plasma creatinine levels were measured by highly specific electron spray ionization (ESI) and tandem LCMS, as described previously (62). Blood pressure was measured using a tail-cuff sphygmomanometer CODA Non-Invasive Blood pressure (Kent Scientific Corporation, CT, USA) using the average of ten independent measures after acclimatization. The plasma intact parathyroid hormone (iPTH) was measured by ELISA kit bought from ALPICO Diagnostics (Salem, NH). Serum calcium, phosphorus, cholesterol, and triglycerides were measured using the Animal Diagnostic Laboratory of the In-Vivo Animal Core at the University of Michigan. We measured the Serum Amyloid A protein using PHASE Murine Serum Amyloid A Assay (SAA; Tridelta, Ireland).

### Atherosclerosis assessment

Each mouse was anesthetized and perfused with PBS through the left ventricle followed by 3 ml of 10% buffered formalin to fix the vascular tree. The aortic tree was removed, micro-dissected to remove adventitial fat, cut longitudinally, stained with Oil Red O (Sigma, St Louis, MO) to visualize neutral lipids, and then pinned on wax plates. The images of the ascending and abdominal aorta were captured on a digital camera, and *en face* plaque quantification of total and lesional surface area was performed with a computerized image analysis program (Image Pro software^,^ Media Cybernetics, Bethesda, MD; (57)). The aortic lesion ratio is derived from the ratio of the Oil Red O stained area to the total surface area of an *en face* section of the aortic tree expressed as a percentage.

### Immunohistochemistry

Aortic root cross sections were cut from paraffin blocks of the aortic root with Leica RM 2155 Microtome and collected on glass slides. Immunohistochemistry was performed on paraffin sections with antibodies against MPO (1:500; Abcam, UK), Mac-2 (macrophage marker; 1:500; Cedarlane, NC), 3-chlorotyrosine (1:1000; Cell Sciences, MA), and 3-nitrotyrosine (1:50; Abcam, UK) along with appropriate negative controls. Biotinylated goat anti-rat IgG and anti-rabbit IgG antibodies (Vector Laboratories, CA) were used as the secondary antibodies. Images were obtained on with Olympus BX-51 microscope and DP-70 high-resolution digital camera (Olympus America Inc, Melville, NY).

### Immunofluorescence and confocal imaging

Snap-frozen aortic sections were incubated with rabbit antibodies for mouse Mac-2, 3-chlorotyrosine, and 3-nitrotyrosine, as detailed above, at 4 °C overnight. Appropriate negative controls were simultaneously processed to assess background autofluorescence. Goat anti-rabbit IgG (Alexa Fluor 555, Jackson Immunoresearch Laboratories, PA) and anti-rat IgG (Alexa Fluor 488) (Cedarlane, NC) were used as secondary antibodies. Dual immunofluorescence labeling was simultaneously scanned by an Olympus FV500 confocal laser-scanning microscope, equipped with a complete integrated image analysis software system (Olympus America Inc, Melville, NY).

### Oxidized amino acid quantification by mass spectrometry

Thoracic aorta was dissected, cleaned, and analyzed as previously described (82) using known concentrations of isotopically labeled internal standards ^13^C_6_ tyrosine, ^13^C_6_ 3-nitrotyrosine, or ^13^C_6_ 3-chlorotyrosine. Oxidized amino acids were quantified by LCMS with ESI with multiple-reaction monitoring MS/MS in positive-ion acquisition mode utilizing an Agilent 6410 triple quadrupole MS system equipped with an Agilent 1200 LC system. All results were normalized for the precursor amino acid, tyrosine. Labeled precursor amino acid, ^13^C_9_^15^N_1_tyrosine, was added to monitor potential internal artifact formation of 3-chlorotyrosine and 3-nitrotyrosine and was noted to be negligible.

### Vascular reactivity experiments

Mouse thoracic aortic rings (about 5 mm above the diaphragm) were mounted in a myograph system (Danish Myo Technology A/S, Aarhus, Denmark), and reactivity was measured as previously reported (83). Force was expressed as a percent of that achieved with 80% of the maximal force after phenylephrine stimulation.

### Statistical analysis

Results are presented as the mean ± standard deviation and a *p*-values <0.05 were considered significant. Differences between the groups were detected using the Student’s *t*-test or Mann–Whitney test (if not normally distributed). For correlation studies, Pearson correlation or Spearman’s correlation (if not normally distributed) was employed. For vascular reactivity studies, data were plotted using sigmoidal interpolation. Maximal steady-state vasodilation values were obtained using nonlinear regression and comparison between groups achieved by Student’s *t*-test. All analyses were made using Graph Pad Prism 7.0 (La Jolla, CA).

## Data availability

All data pertaining to this article are contained within this article and supplemental data.

## Conflict of interest

The authors declare that they have no conflicts of interest with the contents of this article.
