# Supplementary material for: Deletion of bone marrow myeloperoxidase attenuates chronic kidney disease accelerated atherosclerosis
Source: J Biol Chem. 2020 Dec 3;296:100120. doi: 10.1074/jbc.RA120.014095 (PMC7948401; doi:10.1074/jbc.RA120.014095)
Supplement: Figures S1–S4 [file mmc1.pdf]

Supporting Information

Deletion of bone marrow myeloperoxidase attenuates chronic kidney disease accelerated atherosclerosis

Anna V. Mathew[1*], Lixia Zeng[1], Kevin B. Atkins[1], Kiana N. Sadri[1], Jaeman Byun[1], Hideaki Fujiwara[2], Pavan Reddy[2], and Subramaniam Pennathur[1,3*]

[1]Department of Medicine, Division of Nephrology; [2]Department of Medicine, Division of Hematology-Oncology; [3]Department of Molecular and Integrative Physiology; University of Michigan, Ann Arbor, MI, 48105

*Corresponding authors: Subramaniam Pennathur or Anna V. Mathew

Email: spennath@umich.edu or amat@umich.edu

Contents:

Figure S1. Creation of chimeric bone marrow MPO deficiency CKD model.

Figure S2. Bone marrow MPO deficiency in 5/6 nephrectomized mice on a high-fat/ high- cholesterol diet decreases MPO expression in atherosclerotic lesions.

Figure S3. Bone marrow MPO deficiency decreases in MPO oxidative products in atherosclerotic lesions in 5/6 nephrectomized mice.

Figure S4. Bone marrow MPO deficiency decreases atherosclerosis in 5/6 nephrectomized mice.

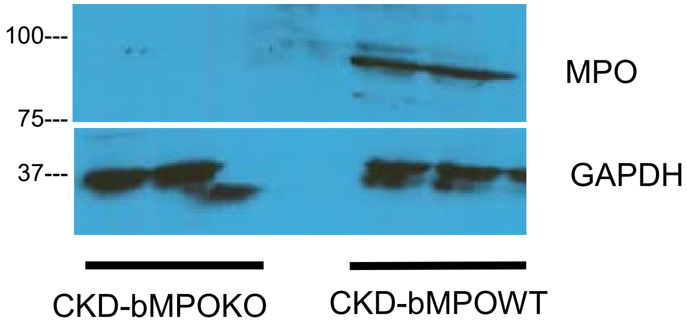

Figure S1. Creation of chimeric bone marrow MPO deficiency CKD model. Immunoblot of bone marrow tissue from irradiated mice with 5/6 nephrectomy (CKD mice) transplanted with bone marrow from MPO knockout mice (CKD-bMPOKO) or WT mice (CKD-bMPOWT). Anti-MPO antibodies (MPO) and anti-glyceraldehyde 3 phosphate dehydrogenase antibodies (GAPDH, loading control) confirmed the absence of MPO in the bone marrow of CKD-bMPOKO mice.

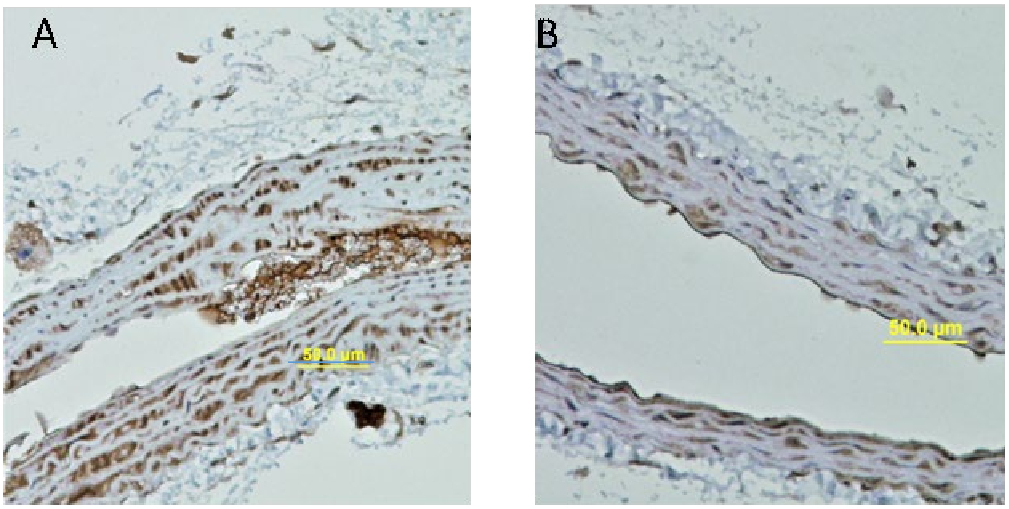

Figure S2. Bone marrow MPO deficiency in 5/6 nephrectomized mice on a high-fat/ high- cholesterol diet decreases MPO expression in atherosclerotic lesions. Representative immunohistologic staining for MPO in aortic cross-sections from CKD mice after 16 weeks on a high-fat/high-cholesterol diet. Intense staining for MPO was observed in the intimal lesions of irradiated, 5/6 nephrectomized mice with WT bone marrow (CKD-bMPOWT, **A**) as compared with mice with MPO-knockout bone marrow (CKD-bMPOKO, **B**). Bars represent 50µm; Original Magnification x20.

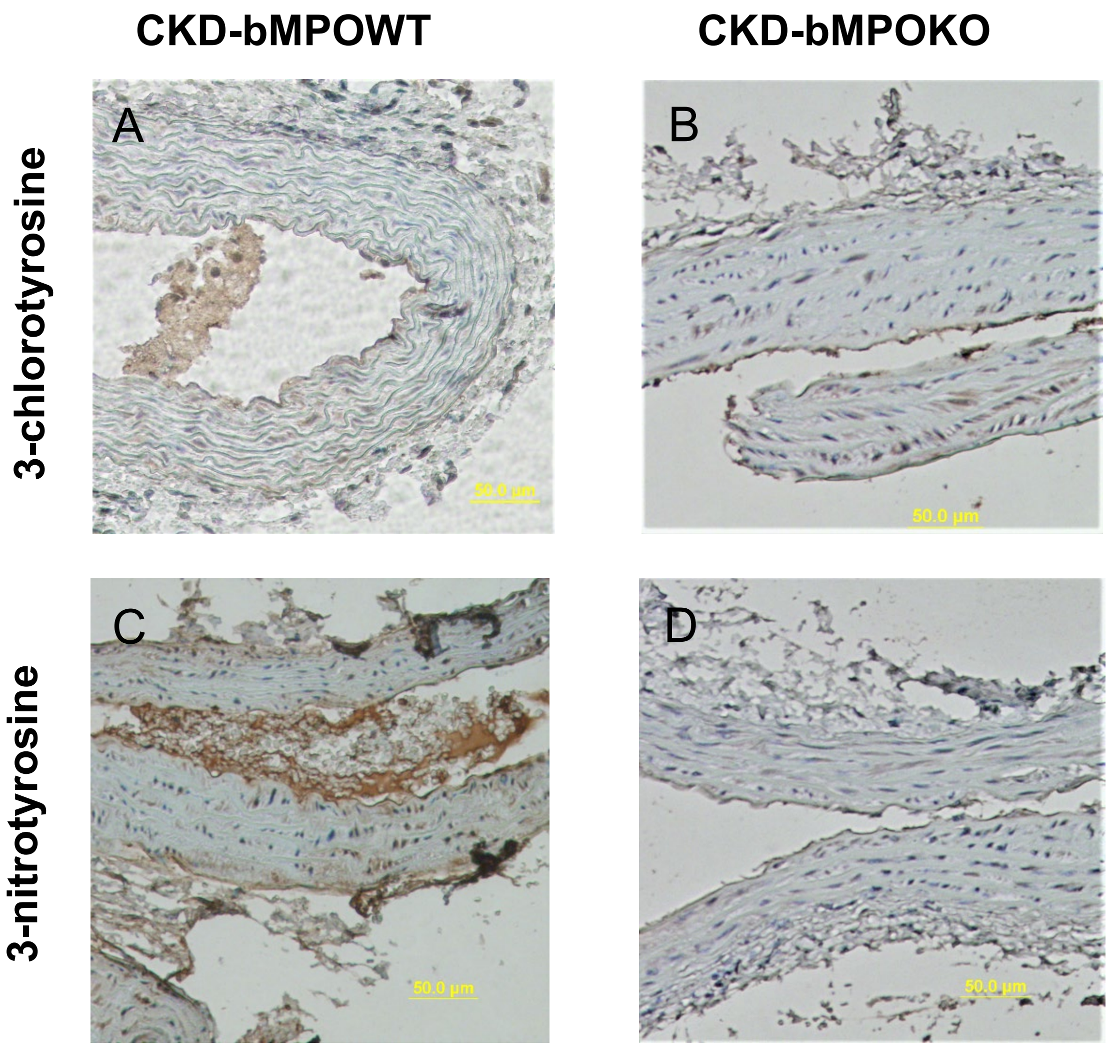

Figure S3. Bone marrow MPO deficiency decreases in MPO oxidative products in atherosclerotic lesions in 5/6 nephrectomized mice. Representative immunohistologic staining for 3-chlorotyrosine (**A, B**) and 3-nitrotyrosine (**C, D**) in aortic cross-sections of mice fed a high-fat/ high- cholesterol diet for 16 weeks. **A and C.** Irradiated CKD mice with WT marrow (CKD-bMPOWT). **B, and D.** Irradiated CKD mice with MPO knockout bone marrow (CKD-bMPOKO). Magnification 20x. Please note intense staining for 3-chlorotyrosine and 3-nitrotyrosine in atherosclerotic intimal lesions of the CKD-bMPOWT mice on a high-fat/ high- cholesterol diet for 16 weeks as compared with age-matched CKD-bMPOKO mice. Bars represent 50µm; Original Magnification x20.

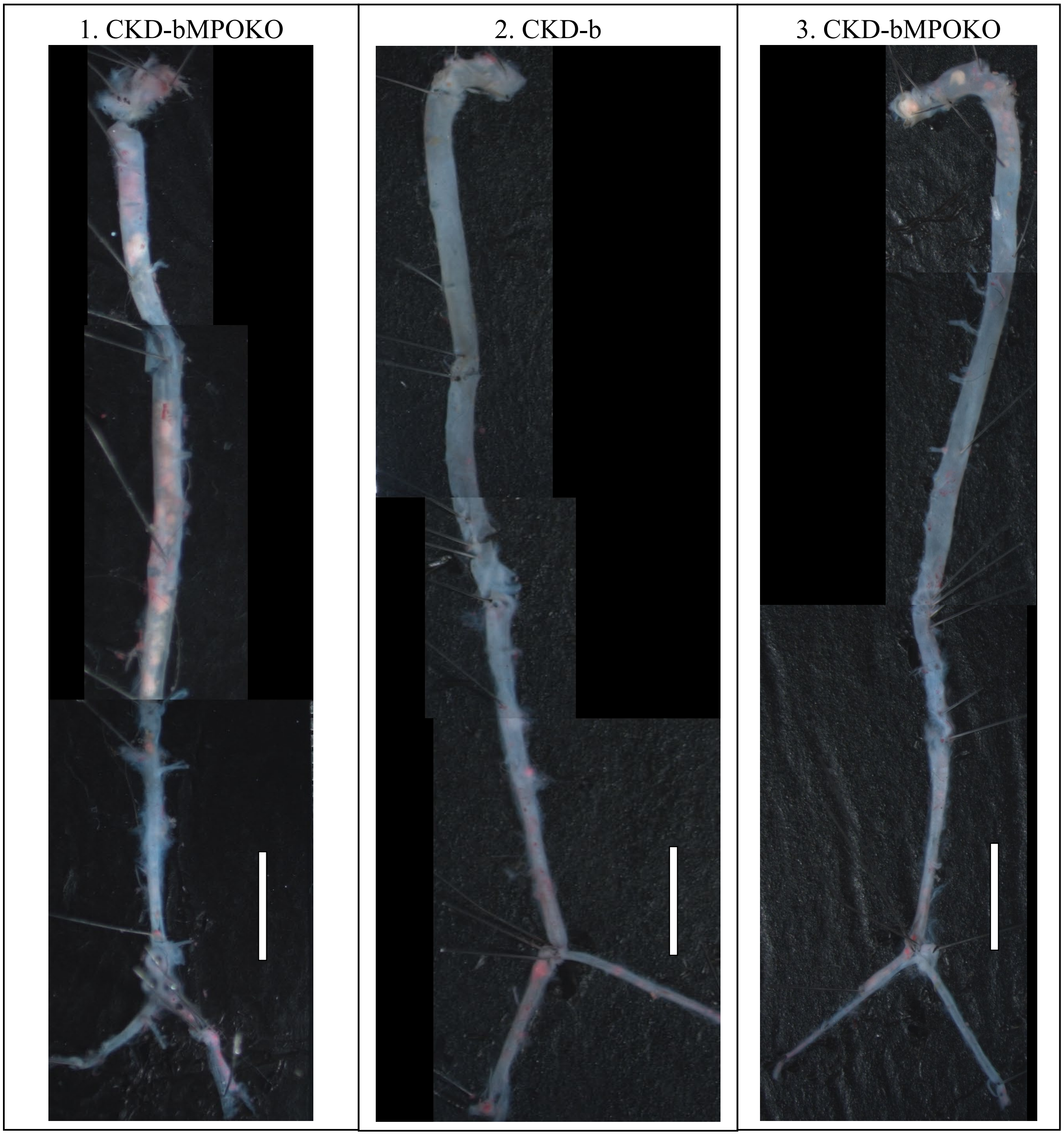
1. CKD-bMPOKO
2. CKD-b
3. CKD-bMPOKO

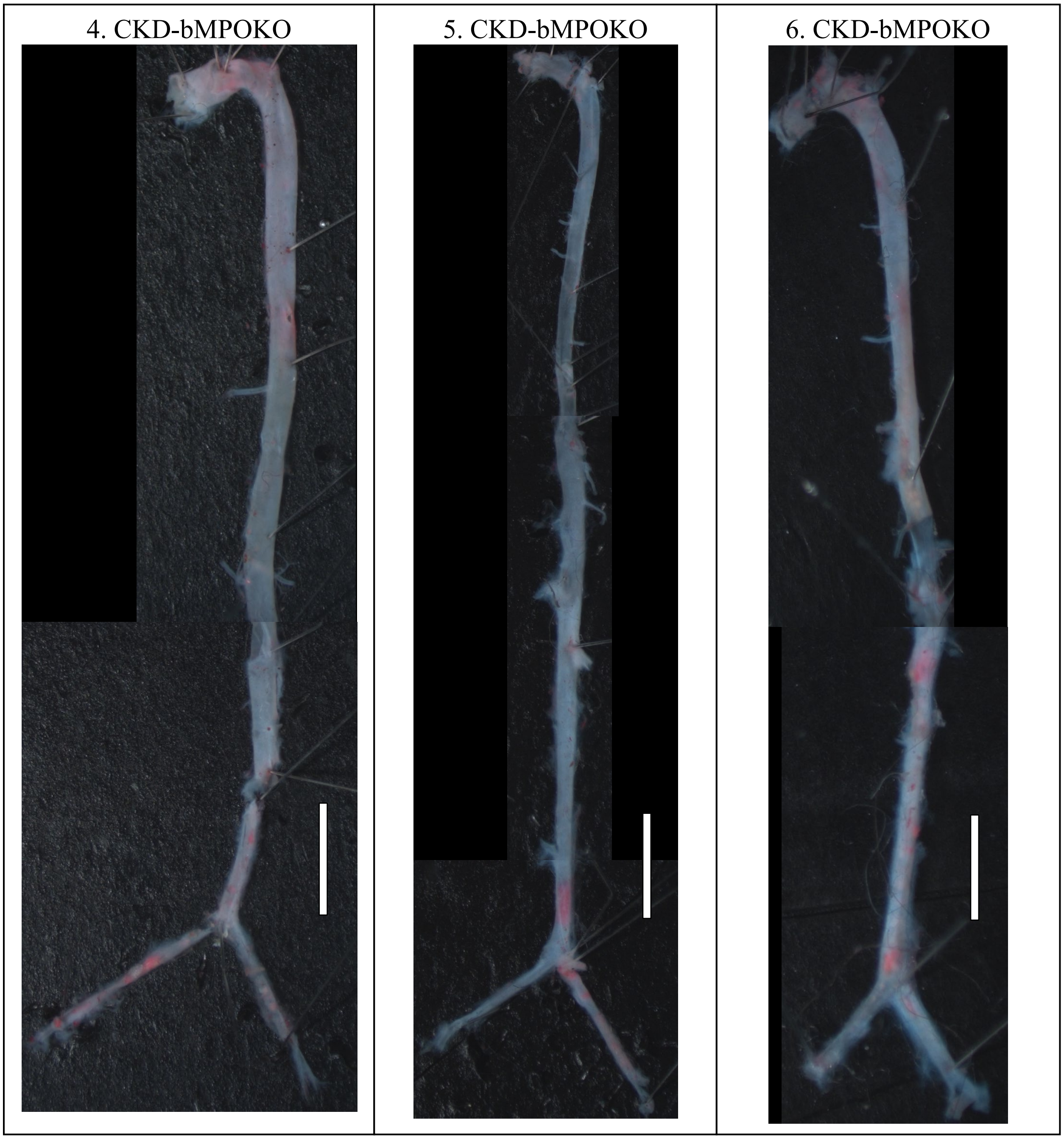
4. CKD-bMPOKO
5. CKD-bMPOKO
6. CKD-bMPOKO

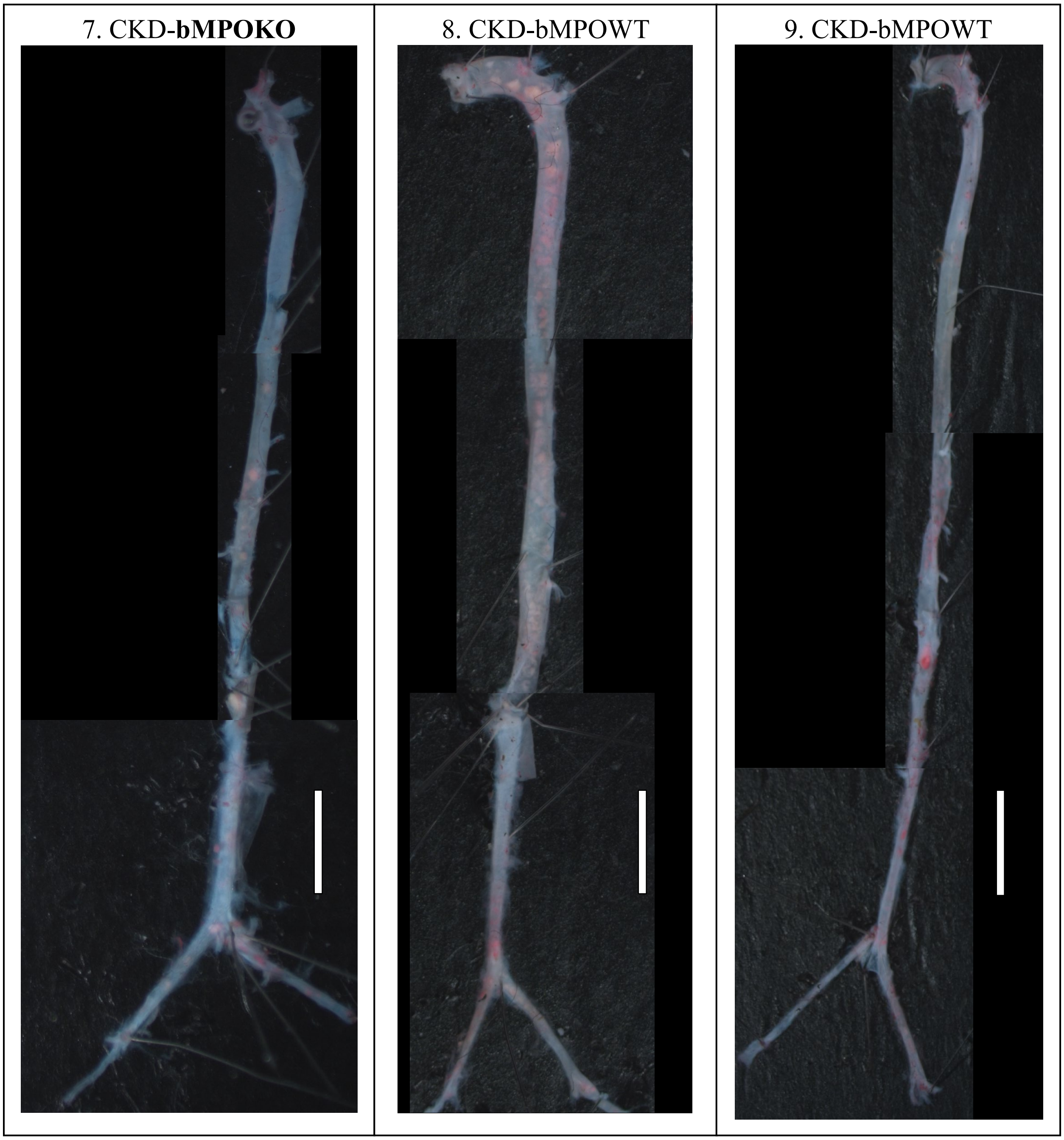
7. CKD-**bMPOKO**
8. CKD-bMPOWT
9. CKD-bMPOWT

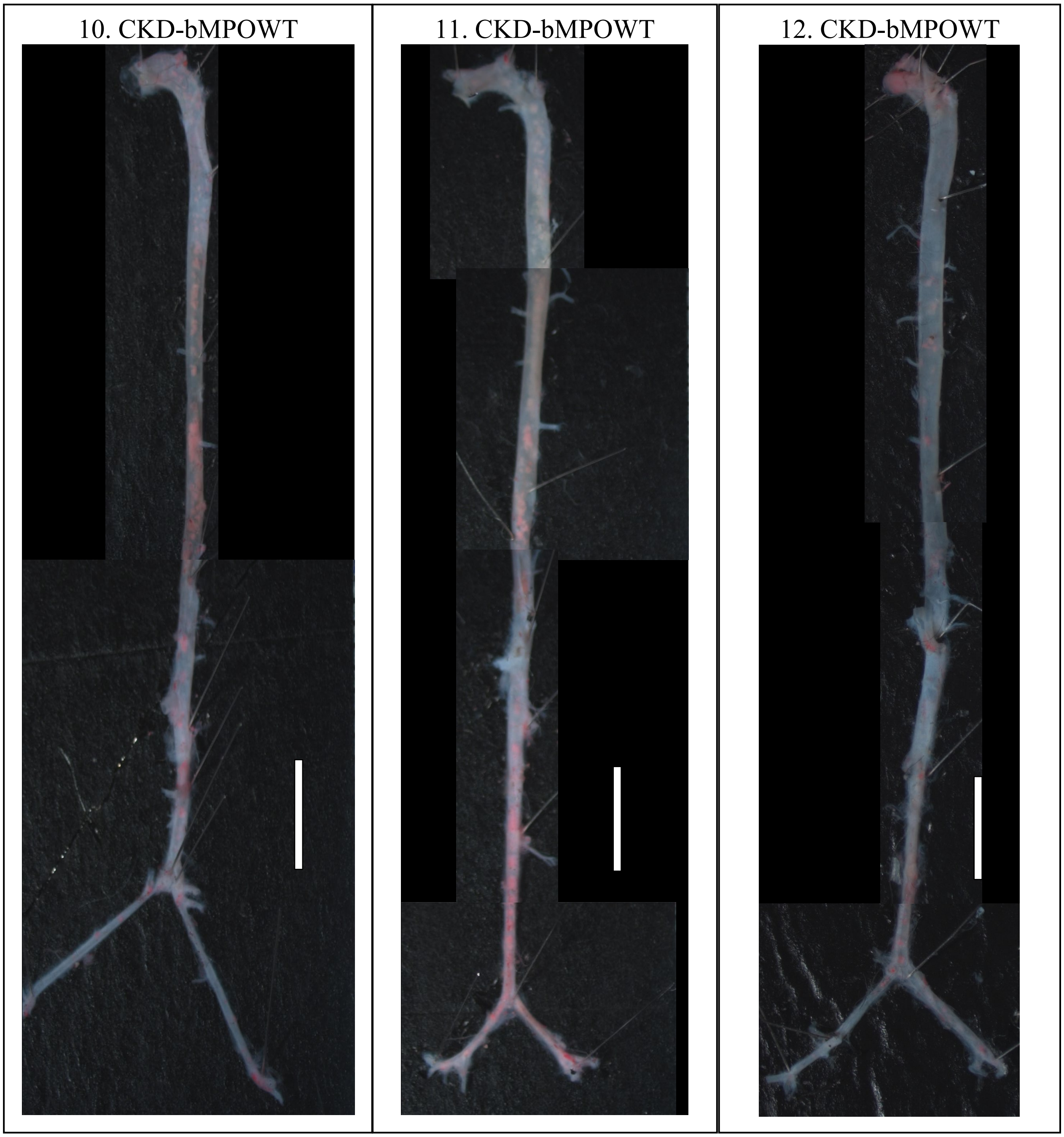
10. CKD-bMPOWT
11. CKD-bMPOWT
12. CKD-bMPOWT

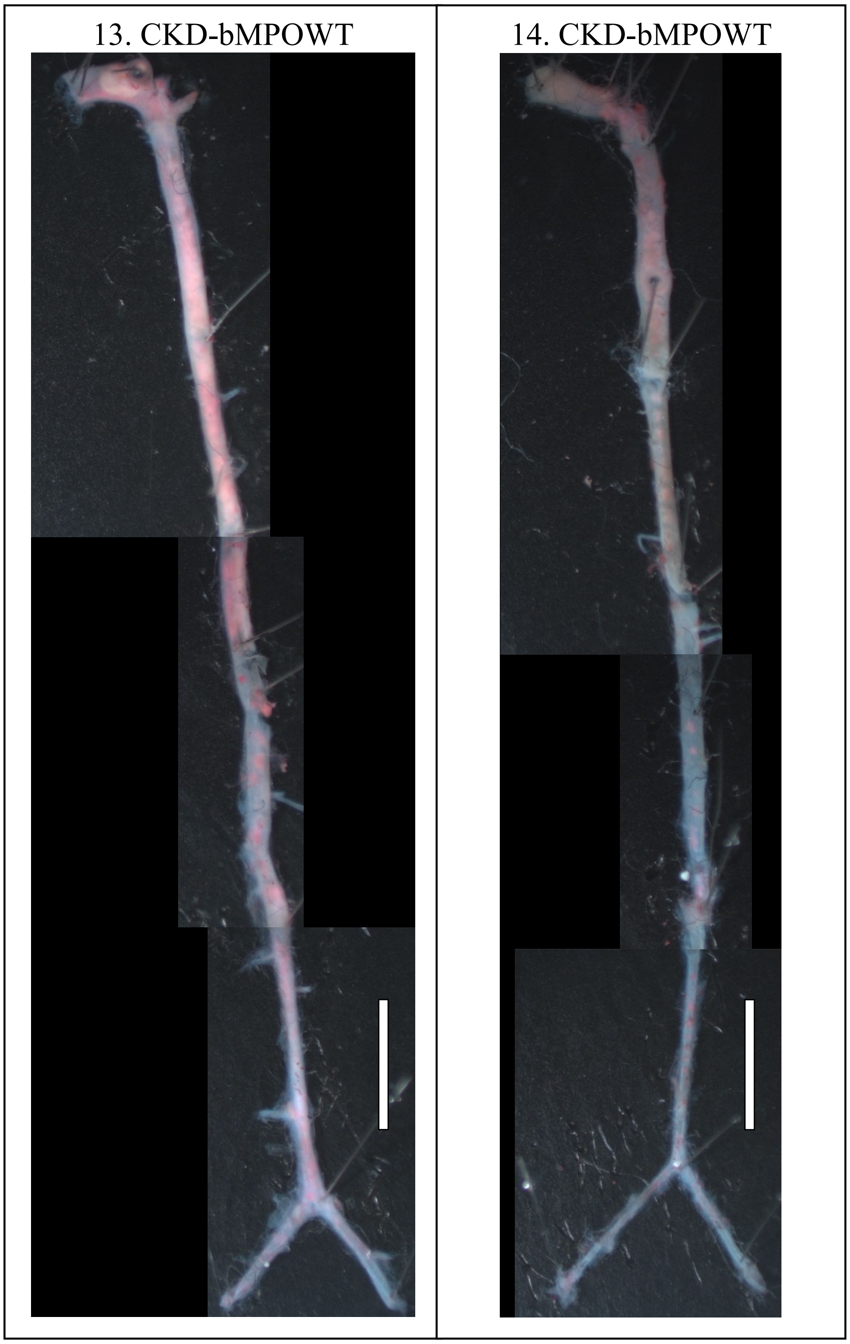

Figure S4. Bone marrow MPO deficiency decreases atherosclerosis in 5/6 nephrectomized mice.

Oil red staining of *en face* aorta from irradiated 5/6 nephrectomized LDLr $^{-/-}$ mice (CKD) after 16 weeks on a high-fat/ high- cholesterol diet represented by composite images. **Panels 1-7.** CKD mice with bone marrow MPO deficiency (CKD-bMPOKO). **Panels 8-14.** CKD mice with WT bone marrow (CKD-bMPOWT). Original Magnification x6.3. Scale bar represents 5mm.
